# Accurate potential energy surfaces for the first two lowest electronic states of the Li (2p) + H_2_ reaction†

**DOI:** 10.1039/c8ra02504e

**Published:** 2018-04-25

**Authors:** Liwei Fu, Dequan Wang, Xuri Huang

**Affiliations:** Laboratory of Theoretical and Computational Chemistry, Institute of Theoretical Chemistry, Jilin University Changchun People’s Republic of China dequan_wang@jlu.edu.cn

## Abstract

The accuracy of three-dimensional adiabatic and diabatic potential energy surfaces is calculated using *ab initio* methods and is numerically fitted for the two lowest electronic states 1 and 2^2^A′ of the LiH_2_ system, which are very important for the Li (2p) + H_2_ reaction. The finite difference method is performed to generate the mixing angles, which are used to educe the diabatic potential from the adiabatic potential. The accurate conical intersection (CI) is studied in this work with three different basis sets. The energy of the conical intersection is slightly lower (nearly 0.12 eV) than that of the perpendicular intermediate on the first excited state. By analyzing the potential energy surfaces in this work we can suggest that the most possible reaction pathway for the title reaction is Li (2p) + H_2_ → LiH_2_ (2^2^A′) (C_2*v*_) → CI → LiH_2_ (1^2^A′) (C_2*v*_) → LiH⋯H → LiH (X^1^*∑*_g_^+^) + H. The conical intersection and (2^2^A′) intermediate may play a vital role in the title reaction.

## Abstract 2

Accurate diabatic potential energy surfaces for the Li (2p) + H_2_ → LiH (X) + H reaction are produced.

## Graphical abstract

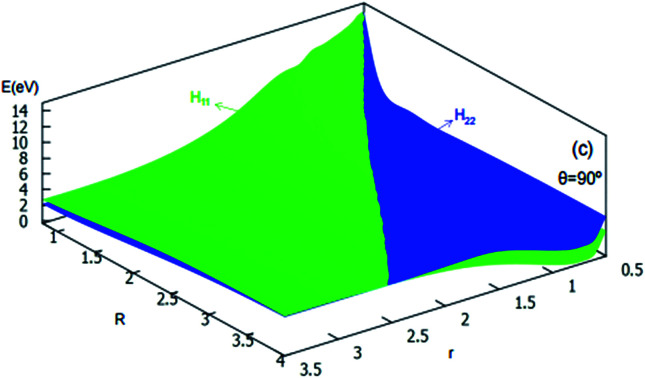

## 1 Introduction

In recent years, lithium chemistry has been thought to play an important role in early cosmic evolution.^1–10^ In the standard Big Bang model, the first star object was formed by gas composed of H, He and Li and some of its isotopes, which means that the chemistry of the early universe is very simple.^11,12^ The formation and depletion of LiH molecules play an important role in stellar evolution and galactic lithium production.^2,3^ The Li + H_2_ reaction is considered as an important pathway of LiH formation.^7,8^

A large number of theoretical studies were essential for identifying and understanding the LiH_2_ system. The investigation of the electronic potential energy surface (PES) was the first essential ingredient. Several 3D-PESs for the ground-state (1^2^A′) of the LiH_2_ system have been well constructed in the past decade^13–18^ and been used for dynamics studies.^19–33^

The reaction, Li (2p) + H_2_ → H + LiH (X), is endoergic by 1624 cm^−1^.^20^ The reaction of Li (2s) + H_2_ → H + LiH (X) on the ground state requires an amount of energy for initiation. Many studies^20,34–36^ proved that Li + H_2_ reactions are considered to follow surface hopping mechanisms by Li insertion into the H–H bond to yield the LiH product. In these conditions the nonadiabatic couplings should be taken into account. The nonadiabatic potential energy surfaces are very interesting for dynamic studies. Up to now, there are two PESs available in the literature that can be used to study non-adiabatic processes for the Li (2p) + H_2_ → H + LiH reaction. The first is the Hsiao and co-workers surface^17^ obtained in 2011. The second one is the He and co-workers surface,^37^ which was investigated in 2016. Hsiao and his co-workers calculated the non-adiabatic PES for the LiH_2_ system at the HF/CAS/MRCI level. In Hsiao’s work, the ground state 1^2^A′ and the lowest excited state 2^2^A′ were calculated using 10a′ and 2a′′ active spaces with the multi-reference configuration interaction (MRCI) method. The potential energies for the excited and ground states have been fitted to the analytical expression in terms of the many-body expansion function. The LiH_2_ configurations were sampled in the ranges *r* = 0.5–7.0 Å, *R* = 0.8–7.0 Å, and *θ* = 1–89° with the Jacobi coordinates (*R*, *r*, *θ*). The work found that the crossing seam lies at the HLiH bending angle *ϕ* = 35–45° and the Li–H distance *R*_3_ = 2.0–2.3 Å while the other LiH distance is fixed at 1.6 Å. Quasi-classical trajectory calculations on the fitted energy function were performed and good results were obtained. He and co-workers^37^ performed a global diabatic PES, which was correlated with the ground state 1^2^A′ and the first excited state 2^2^A′ of the Li (2p) + H_2_ reaction. These potential energies were calculated with two regions, the Li–H_2_ reactant region and the H–LiH product region. The energies were scanned in the ranges *R*_Li–HH_ = 0.0–30.0*a*_0_, *r*_HH_ = 0.6–30.0*a*_0_, *θ* = 0.0–90.0°, and *R*_H–LiH_ = 0.0–30.0*a*_0_, *r*_LiH_ = 1.3–30.0*a*_0_, *θ* = 0.0–90.0° for the reactant region and the product region, respectively. Then the authors converted the adiabatic energies to diabatic potential energies and fitted the diabatic potential energies by the NN method. In this way, an accurate global diabatic PES was performed. In the present work, the authors calculate the LiH_2_ adiabatic potential energies in a larger range and with more data points than before and use a more accurate fitting method to improve the PES level. The conical intersection point is accurately studied too.

The main differences between the present work and former work are as follows: firstly, the atom distances of *r*(H–H) and *R*(Li–HH) are scanned to 32 Å, which is larger than those done before and ensures that all of the dynamic studies are in the accurate scan area; secondly, a more accurate fit method, the three dimensional B-spline method, is used to make sure that accurate PESs are obtained; thirdly, many more geometries (83 930) are generated for every adiabatic potential energy.

The outline of the present work is as follows. The second chapter introduces the calculation method for the adiabatic energies. The adiabatic and diabatic PESs of LiH_2_ are presented in the third chapter. The fourth chapter shows a simple summary for the present work.

## 2 Computational methods

In this work, the *ab initio* calculations have been carried out at the HF/MSSCF/MRCI level with the MOLPRO 2012 package^38^ using the large basis sets (aug-cc-pV5Z) and a full-valence active space involving five valence electrons in ten orbitals was employed in the MSSCF procedure. Furthermore, one 1s orbital of the lithium atom was kept doubly occupied. In the subsequent MRCI calculations, one 1s orbital of the lithium atom was frozen. In this system, we use the Jacobi coordinates (*r*, *R*, *θ*) to characterize this three-body system, in which *r* indicates the bond length of the two hydrogen atoms, *R* shows the distance of the lithium atom from the center of mass of the two hydrogen atoms, and *θ* represents the angle between the *r* an *R* vectors. We sampled the LiH_2_ configurations in the ranges *r* = 0.4–32 Å, *R* = 0.0–32 Å, and *θ* = 0.0–90.0°. For *θ* = 0.0–30°, the angle grid is 5°. 164 *r* points and 55 *R* points were used with different step sizes and 63 140 geometries were chosen to generate the *ab initio* energy points in this region. For *θ* = 40.0–90.0°, the angle grid was enlarged to 10° and 63 *r* points and 55 *R* points were used, so 20 790 geometries were achieved here. A total of 83 930 geometries were generated for every adiabatic potential energy. These large number points warrant the quality of the following fitting PESs. Inside the whole scan field the procedure interpolates the surfaces using the three dimensional B-spline method.

Considering the two coupling states of LiH_2_, the diabatic energies ***H***^d^_*ii*_ can be obtained in terms of our fitted adiabatic energies ***E***^a^_*i*_ by

1 ***H***^d^_11_ = cos^2^ *α****E***^a^_1_ + sin^2^ *α****E***^a^_2_;

2 ***H***^d^_22_ = sin^2^ *α****E***^a^_1_ + cos^2^ *α****E***^a^_2_;

3 ***H***^d^_12_ = cos *α* sin *α*(***E***^a^_2_ − ***E***^a^_1_);

4 ***H***^d^_12_ = ***H***^d^_21_.

***H*** ^d^_11_ and ***H***^d^_22_ are the corresponding diabatic energies for the diabatic PES; ***H***^d^_12_ and ***H***^d^_21_ are the coupling potential energies between the two diabatic states. The mixing angle *α* was obtained by the finite difference method^39–43^ using the Molpro program.

## 3 Results

In this work, we calculated the three dimensional adiabatic and diabatic PESs for the LiH_2_ system. For easy to understand and discuss these PESs, we regard the ground state energy of the Li (2s) atom to be 0 eV, far away from that of the equilibrium structure of H_2_.

### 3.1 Diatomic atom potential energy

**Fig. 1:**
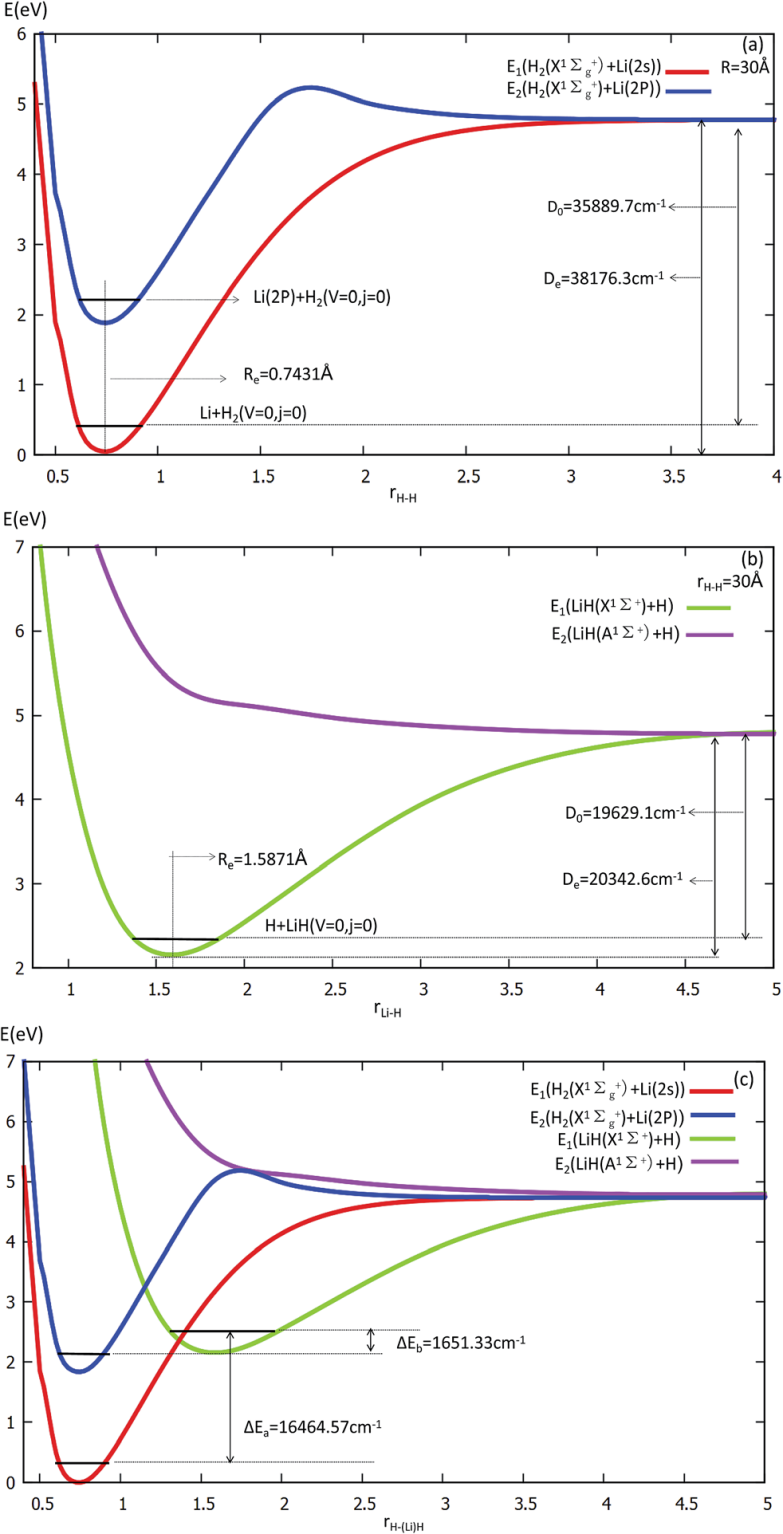
Potential energy surfaces (in eV) are plotted as a function of the distances *r*(H–H) and *r*(Li–H) (in Å) at the angle *θ* = 0° in Jacobi coordinates.

The equilibrium structures and electronic energies for the diatomic molecules (H_2_ and LiH) are derived from our fitting PES. To obtain the H_2_ PES, we fixed *R* = 30 Å and *θ* = 90.0°. For deriving the LiH PES, we fixed *r* = 30 Å and *θ* = 0.0°. The detailed results are shown in Fig. 1 and listed in Table 1. Our fitting PESs display that the difference of the Li (2s) and Li (2p) energies is 14 803.2 cm^−1^. From panel (a) in Fig. 1, one can see that the equilibrium distance (*R*_e_) of H_2_ is 0.7431 Å and the dissociation energy (*D*_e_) is 35 889.7 cm^−1^, which are in good agreement with the experimental results of 0.74144 Å (ref. 45) and 36 118.06 cm^−1^,^46^ respectively.

**Table 1:** Spectroscopic data for H_2_ and LiH

Species	Parameter	This work	Lee’s work^a^	Experimental
Li	Δ*E* (2p ← 2s) [cm^−1^]	14 803.2	14 914	14 904^b^
H_2_ (X^1^*Σ*_g_^+^)	*R*_e_ [Å]	0.7431	0.743	0.7414^c^
	*D*_e_ [cm^−1^]	38 176.3	37 868	38 288^c^
	*D*_0_ [cm^−1^]	35 889.7	35 687	36 118.06^d^
LiH (X^1^*Σ*^+^)	*R*_e_ [Å]	1.5871	1.60	1.5956^e^
	*D*_e_ [cm^−1^]	20 342.6	19 705	20 287.7^e^
	*D*_0_ [cm^−1^]	19 629.1	19 011	19 589.8^e^

a Ref. 20.

b Ref. 44.

c Ref. 45.

d Ref. 46.

e Ref. 47.

Panel (b) in Fig. 1 exhibits that *R*_e_ = 1.5871 Å and *D*_e_ = 19 629.1 cm^−1^ for LiH. These are in good accordance with Stwalley’s experimental results of 1.59558 Å and 19 589.8 cm^−1^,^47^ respectively.

Energy curves for the two lowest states of the H_2_ and LiH are shown at panel (c) in Fig. 1.

From panel (a) one can see that the curve of the first excited state includes two parts of the PES, *i.e.* H_2_ (singlet) + Li (2p) and H_2_ (triplet) + Li (2s). When *r*(H–H) becomes larger, the energies of H_2_ (triplet) + Li (2s) and that of H_2_ (singlet) + Li (2s) are degenerate, so the two curves approach the same point. This characteristic is also suited to the LiH PES curve. As shown in panel (c), when the three atoms are far away from each other, the energies of every curve approach the same point.

### 3.2 Three-dimensional adiabatic potential energy surfaces

**Fig. 2:**
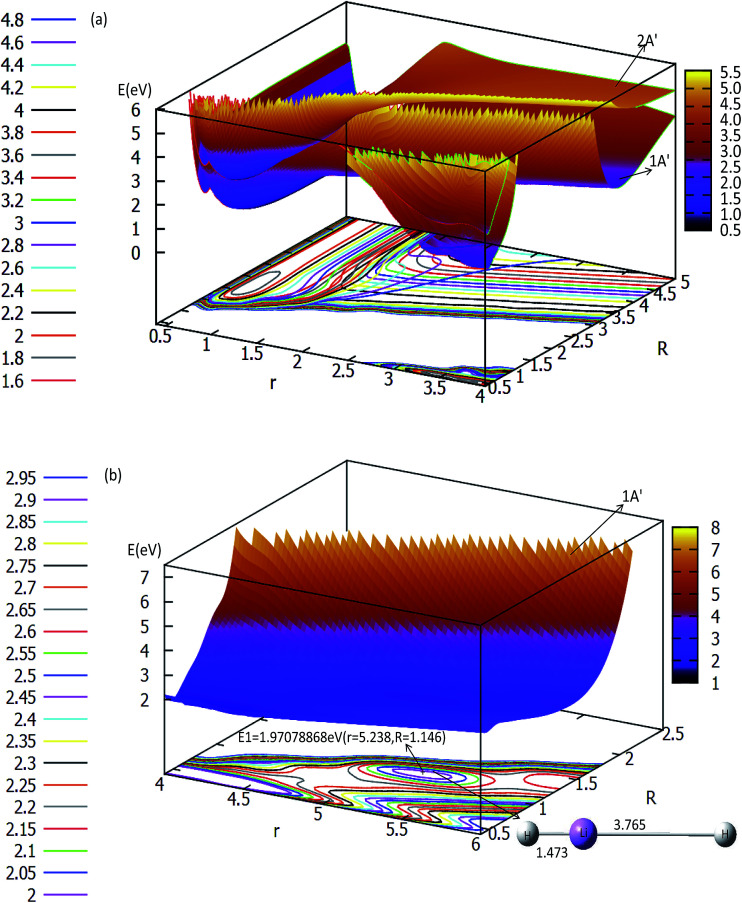
Two potential energy surfaces (in eV) and contour plots of the potential energy surface as a function of distances *r* and *R* (in Å) at the angle *θ* = 0° in Jacobi coordinates.

**Fig. 3:**
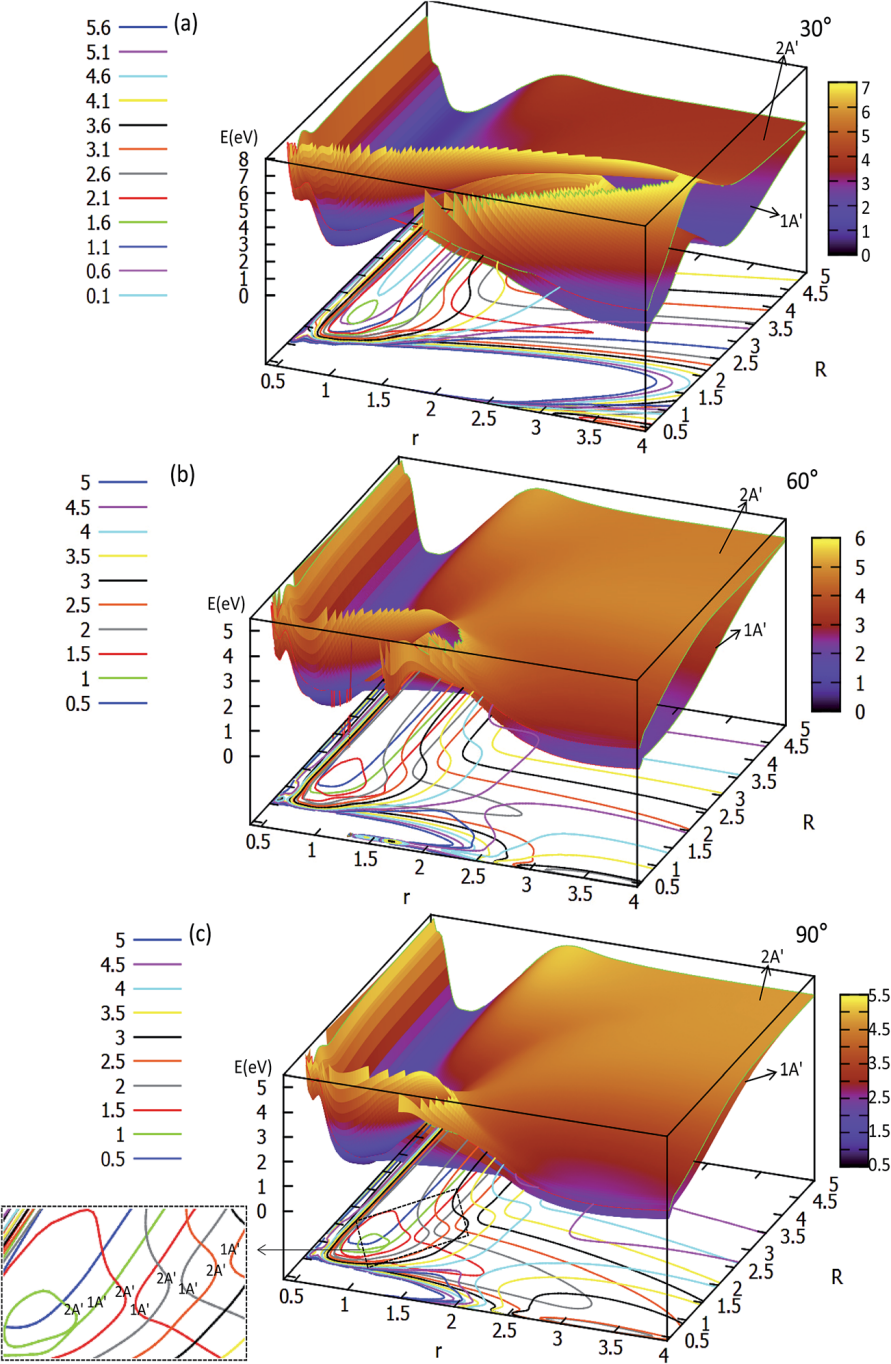
Two potential energy surfaces (in eV) and contour plots of the potential energy surface as a function of distances *r* and *R* (in Å) for the different angles *θ* = 30°, 60° and 90°.

The three-dimensional adiabatic potential energy surfaces (3D-PESs) of LiH_2_ are plotted in Fig. 2, 3 and Fig. S1A–6A† for *θ* = 0.0°, 15.0°, 30.0°, 60.0°, and 90.0°, respectively.

The panel (a) in Fig. 2 is a multi plot of the ground state LiH_2_ (1^2^A′) and the first excited state LiH_2_ (2^2^A′) PESs with *θ* = 0°. The separated figures are plotted in panels (a) and (b) in Fig. S1A.† From panel (a) in Fig. 2 and panel (a) in Fig. S1A†, one can find that for the ground state reaction, the entrance, which is the reaction starting from the reactants, of Li (2s) + H_2_ has no reaction barrier, and the PES has no minimum geometries. But for the lowest excited state of LiH_2_ (2^2^A′) there is a minimum when *θ* = 0.0° (see panel (a) in Fig. 2 and panel (b) in Fig. S1A†). The corresponding geometry of this minimum is *r*(H–H) = 0.741 Å and *r*(LiH) = 1.838 Å, and the energy of this structure is 0.1686 eV lower than that of the entrance for this linear structure. Panel (b) in Fig. 2 is enlarged, plotting the product part (LiH (X^1^*Σ*_g_^+^) + H). This panel reveals that first a Li–H⋯H complex is formed in the ground state reaction pathway, then after a reaction barrier the system reaches the LiH (X^1^*Σ*_g_^+^) + H product. The geometry of this collinear complex is *r*(Li–H) = 1.473 Å and *r*(H–H) = 3.765 Å, and the corresponding energy is 1.9708 eV higher than that of the entrance.

The ground state and the first excited state of the LiH_2_ PESs for *θ* = 15° are plotted in Fig. S2A† and Fig. S3A.† The features of these PESs are similar to that of *θ* = 0°. Firstly, there is a minimum structure on the first excited PES (see panel (a) in Fig. S2A† and panel (d) in Fig. S3A†), and its energy is 1.618 eV higher than that of the ground state entrance, and it is 0.2191 eV lower than that of the entrance of the excited state. Secondly, there is a product complex in the ground state PES, and the energy of this complex is 2.0830 eV higher than that of ground state entrance. The product complex for this *θ* = 15° is 0.1122 eV higher than that of *θ* = 0°. This result indicates that the production is in the small *θ* range. For obtaining the accurate PESs of the product part, a small grid both for the angle and for the distance is used to scan the energies for this area.

The two lowest adiabatic PESs of LiH_2_ for *θ* = 30° and *θ* = 60° are plotted in panels (a) and (b) in Fig. 3, respectively. The characteristics of the PESs for these two angles are different from that of 0° and 15°, *i.e.* there is no product complex in these PESs. The consistent feature of these four angles PESs is that there is a minimum in the first excited state. The minimum energies are 0.311 eV and 0.747 eV lower than those of the entrance for 30° and 60°, respectively. The detailed minimum geometry is shown in panel (b) in Fig. S4A† and Fig. S5A.†

**Fig. 4:**
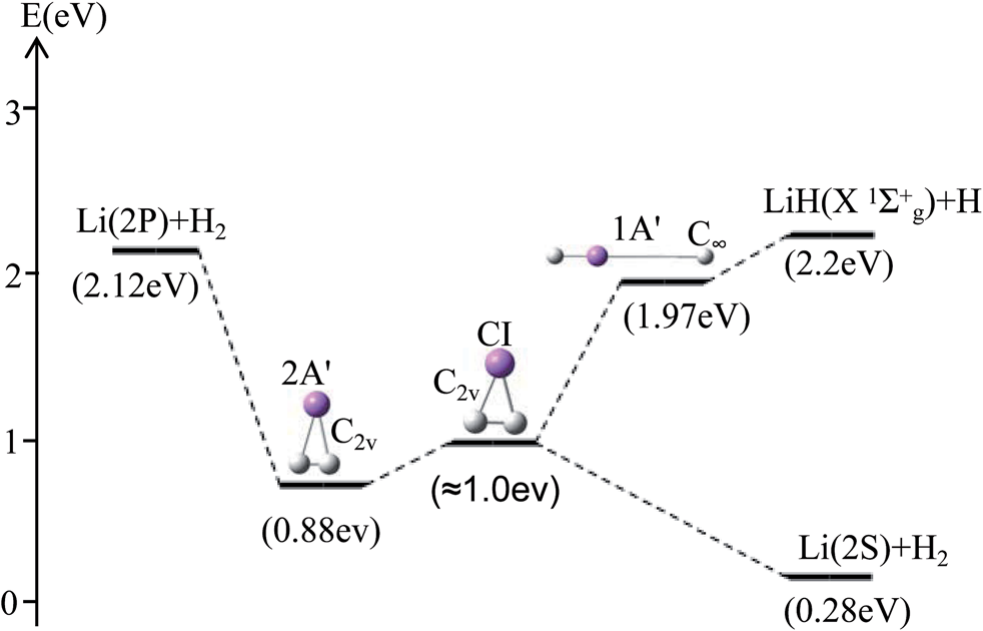
The most possible reaction pathway for the Li (2P) + H_2_ → LiH (X^1^*Σ*_g_^+^) + H reaction.

For *θ* = 90° the PESs of the lowest two states of LiH_2_ are multi plotted in panel (c) in Fig. 3 and the separated plot is shown in panels (a) and (b) in Fig. S6A.† As with the other angle PESs, there is no minimum on the ground state and there is one minimum on the first excited state. The energy of this minimum is 0.975 eV lower than that of the entrance energy. The energy of this minimum is the lowest in the excited state, so it is the global minimum for the first excited state. From the contour plot of the lowest two PESs of LiH_2_ (see panel (c) in the Fig. 3), the 1 eV energies are shown with the green ring and the adjacent green curve contour line for (2^2^A′) and (1^2^A′) LiH_2_, respectively. These two lines are nearly coinciding when the geometries are in the ranges *r* ≈ 1.0 Å and 1.5 < *R* < 2.0 Å. The 2 and 1^2^A′ states lie very close to each other in these geometries. So the conical intersection for the title system is in this area. In other words, the surface transition from the 2^2^A′ to 1A′ state frequently occurs in this area. According to the above discussions, we can come to the conclusion that the most possible reaction pathway (see Fig. 4) for Li (2p) + H_2_ → LiH (X) + H is as follows: firstly, the lithium atom attacks H_2_ to form the perpendicular (C_2*v*_) LiH_2_ (2^2^A′) intermediate; secondly, the system passes the conical intersection and the electron transits from the 2^2^A′ to 1^2^A′ surface; and lastly, LiH_2_ (1^2^A′) separates into two parts product LiH (X) + H. Comparing Fig. 1 and 3 one can see that the energy of the conical intersection (nearly 1 eV) is lower than that of Li (2P) + H_2_ (nearly 2 eV) (hence no reaction barrier), so the required kinetic energy for the Li (2p) + H_2_ → LiH (X) + H reaction is just the endothermic energy of the reaction. That means that the reaction starts from the reactants Li (2P) + H_2_ and proceeds through the most possible reaction pathway to reach the products LiH (X) + H. There is no higher energy transition state, the only collision energy needed is the difference in energy between the products and reactants, *i.e.* the endothermicity (1624 cm^−1^) of the reaction.

### 3.3 Three dimensional diabatic potential energy surfaces

#### (a) Conical intersection

**Fig. 5:**
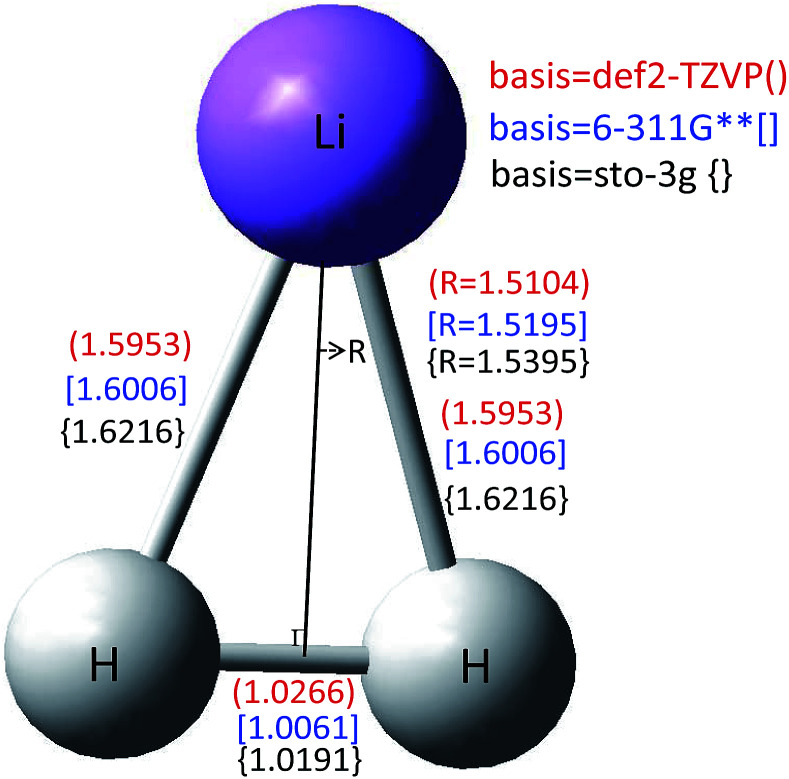
The conical intersection structure for the 1 and 2^2^A′ of LiH_2_ calculated with three different basis sets.

The geometry of the conical intersection for the 2 and 1^2^A′ states of the title system are calculated using the CASSCF method with three different basis sets, *i.e.* def2-TCVP, 6-311G**, and sto-3g. All of the data calculated with these three basis sets are conformed to each other. The detailed structure is shown in Fig. 5. Fig. 5 reveals that the geometry of the conical intersection is C_2*v*_ symmetry, and the bond length of *r*(H–H) is nearly 1.0 Å, and *R*(Li–HH) is nearly 1.5 Å. This is in good agreement with our results (see Fig. 3).

#### (b) Mixing angles

**Fig. 6:**
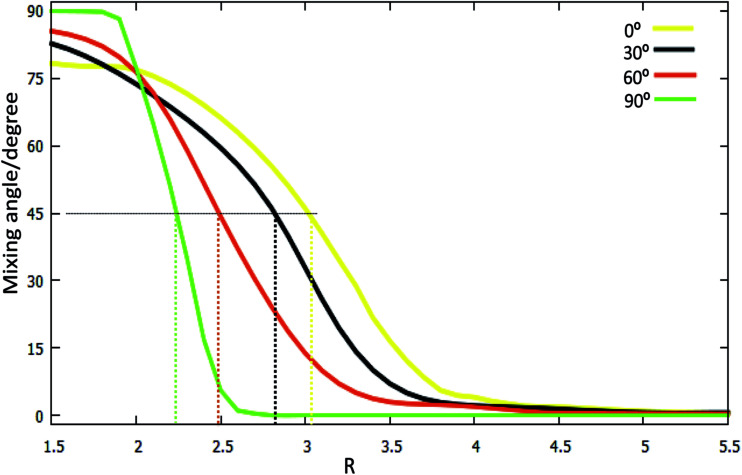
The mixing angles (*α* [in degree]) as a function of *R* (in Å) for fixed *r* = 1.4 (in Å) at the different angles *θ* = 0°, *θ* = 30°, *θ* = 60°, *θ* = 90° in Jacobi coordinates.

A part of mixing angles, which can be employed to construct diabatic potentials from the adiabatic potentials, are shown in Fig. 6. When the mixing angle *α* = 90°, the relationship between adiabatic energy (*E*_1_ and *E*_2_) and diabatic energy (*H*_11_ and *H*_22_) is *E*_1_ = *H*_11_, *E*_2_ = *H*_22_; when *α* = 0°, *E*_1_ = *H*_22_ and *E*_2_ = *H*_11_; the cross point appears when the mixing angle *α* = 45°. The diabatic potential for the other mixing angle can be calculated with formulas (1)–(4).

#### (c) Diabatic potentials

**Fig. 7:**
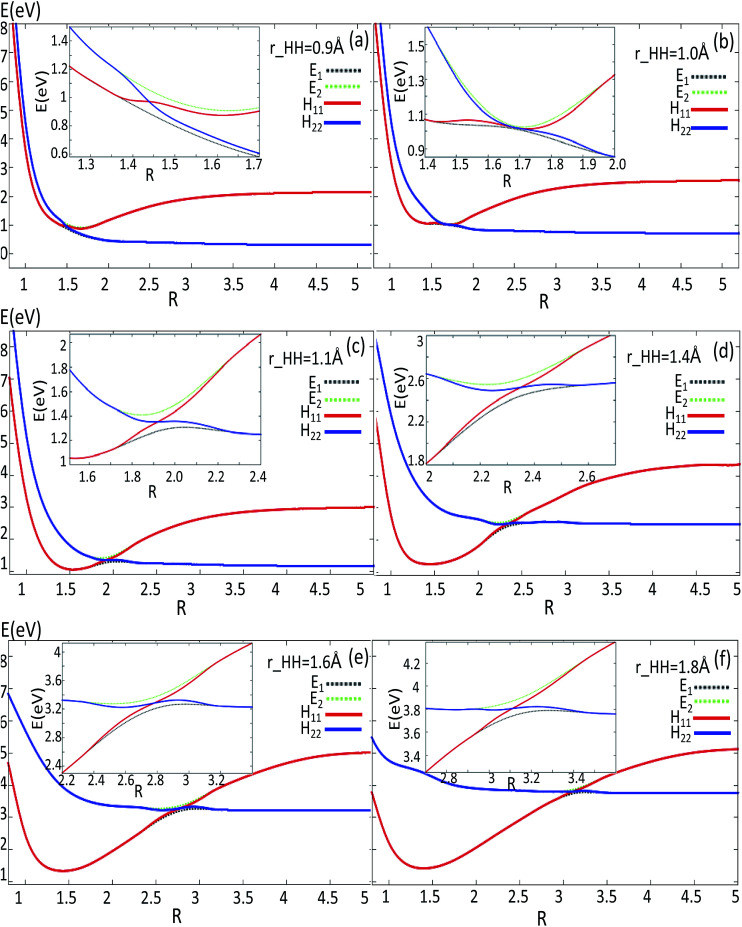
Adiabatic and diabatic potentials (in eV) as a function of distance *R* (in Å) for the fixed angles *θ* = 90° at *r* = 0.9 Å, *r* = 1.1 Å, *r* = 1.4 Å, *r* = 1.6 Å, *r* = 1.8 Å in Jacobi coordinates.

**Fig. 8:**
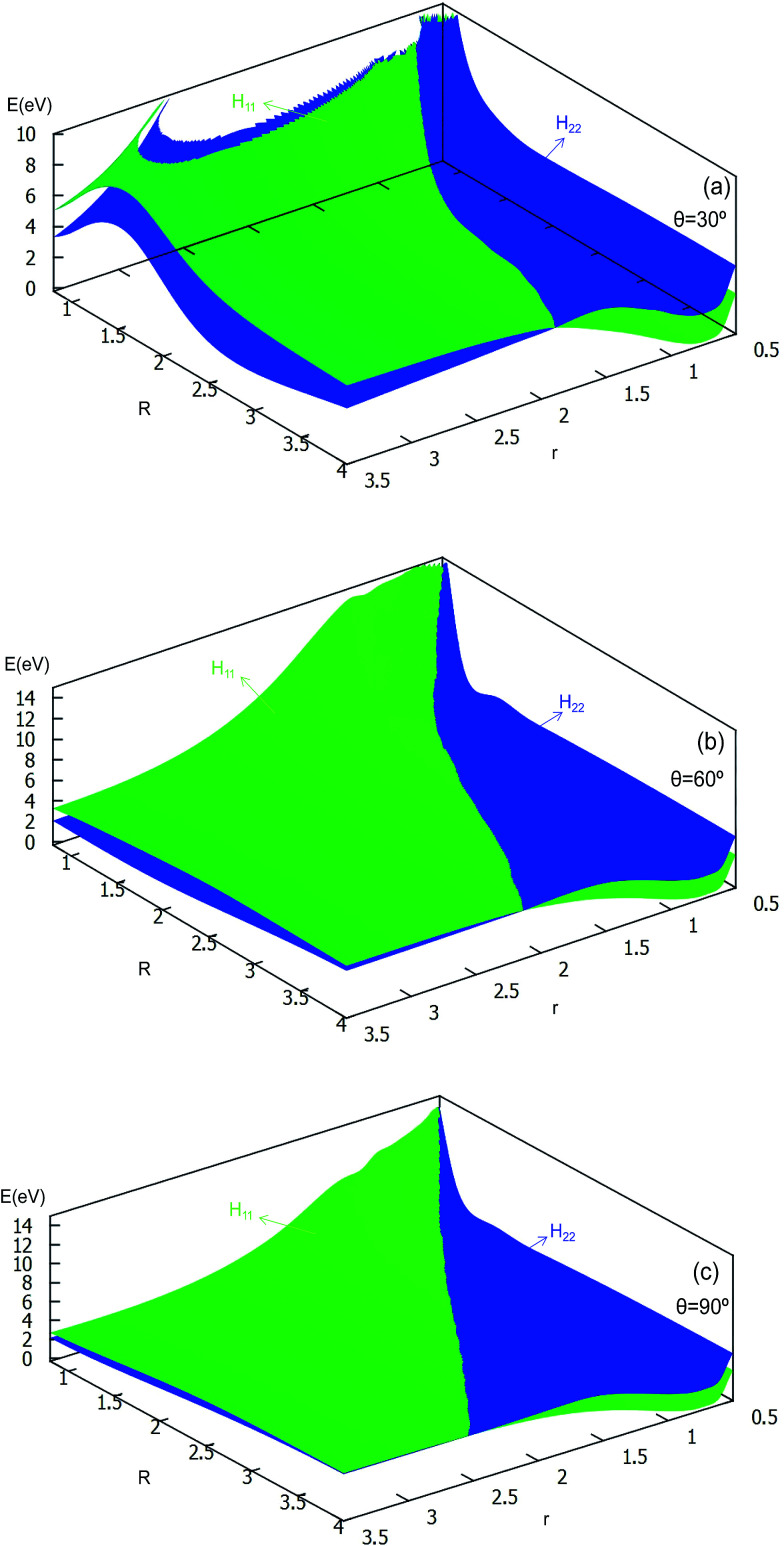
Diabatic potential energy surfaces (in eV) as a function of distances *r* and *R* (in Å) at the different angles *θ* = 30°, *θ* = 60°, *θ* = 90° in Jacobi coordinates.

The global diabatic potentials are derived using formulas (1)–(4). To show clearly these diabatic potentials, the 1D and 2D adiabatic and diabatic potentials are plotted in Fig. 7 and 8, respectively. Because the angle *θ* for the conical intersection is 90 degrees, the 1D diabatic and adiabatic potentials are plotted with different *r*(H–H) values for this angle. The area near the diabatic potential cross point is enlarged and plotted in the same panel. Fig. 7 reveals that when *r* = 0.9 Å (panel (a)), the distance *R* is shorter than that of the minimum; when *r* = 1.0 Å (panel (b)), which is near the conical intersection geometry, the cross point is nearly at the minimum of both of the two state potentials; when *r* > 1.0 Å (panels (c)–(f)), the *R* for the cross point is larger than that of the minimum of the two states. Comparing all the panels in this figure, the *R* for the cross point increases with the increasing of the *r* length.

The 2D diabatic potentials for *θ* = 30°, 60°, and 90° are plotted in Fig. 8. In this figure, the green surfaces describe the *H*_11_ potential, and the blue surfaces show the *H*_22_ potential. According to these three panels one can conclude that when the two hydrogen atoms are at a short distance the ground state of the title system is *H*_11_ potential. If the distance of the two hydrogen atoms is increased (the title system after the cross point), the *H*_22_ potential energy are the ground state energy. Furthermore, these three panels exhibit that the cross points are nearly on the same line for each angle.

## 4 Conclusions

We present *ab initio* calculated adiabatic potential energy surfaces of the ground state (1^2^A′) and the first excited state (2^2^A′) for the LiH_2_ system by the MOLPRO quantum chemistry package in Jacobi coordinates. In total 83 930 geometries were used to generate every state’s adiabatic potential, so an accurate and larger region of configuration space for the potential energy surfaces for the ground and the first excited states is produced. For obtaining the accurate diabatic potentials, mixing angles were derived with a finite difference method. The conical intersection geometries for the two lowest states were also studied in this work with three different basis sets.

Our work has predicted some essential features of these two lowest states. There is a global minimum, *i.e.* a perpendicular (C_2*v*_) intermediate, on the first excited state LiH_2_ (2^2^A′) potential. The conical intersection appeared near the intermediate, and the energy of this conical intersection is slightly higher (≈0.12 eV) than that of the intermediate. Comparing Fig. 1 and 3 one can see that the energy of the conical intersection (nearly 1 eV) is lower than that of Li (2P) + H_2_ (nearly 2 eV) (hence no reaction barrier), so the required kinetic energy for the Li (2p) + H_2_ → LiH (X) + H reaction is just the endothermic energy of the reaction. That means, the reaction starts from the reactants Li (2P) + H_2_, proceeds through the most possible reaction pathway to reach the products LiH (X) + H, and there is no higher energy transition state. The only collision energy needed is the difference in energy between the products and reactants, *i.e.* the endothermicity (1624 cm^−1^) of the reaction. There is a complex LiH⋯ H with angle *θ* = 0° on the 1^2^A′ potential. So the most possible reaction pathway for Li (2P) + H_2_ → LiH + H is as follows: firstly, the Li (2P) atom attacks H_2_ and forms a perpendicular (C_2*v*_) LiH_2_ (2^2^A′) intermediate; secondly, the intermediate passes the conical intersection reaching the LiH_2_ (1^2^A′) potential; thirdly, a partial electron of the lithium atom transfers to one hydrogen atom, then the Li–H bond is formed and the H–H bond is broken to form a LiH⋯H complex; lastly, the complex separates into the LiH (X^1^*Σ*_g_^+^) + H product.

It is worth performing the full dynamical study with these global diabatic potential energy surfaces. We will continue this work in the following study. We will make sure that in the following work we can obtain more interesting results.

## Conflicts of interest

There are no conflicts to declare.

## Supplementary Material

RA-008-C8RA02504E-s001
