# Supplementary material for: Accurate potential energy surfaces for the first two lowest electronic states of the Li (2p) + H2 reaction
Source: RSC Adv. 2018 Apr 25;8(28):15595–602. doi: 10.1039/c8ra02504e (PMC9080088; doi:10.1039/c8ra02504e)
Supplement: RA-008-C8RA02504E-s001 [file RA-008-C8RA02504E-s001.pdf]

Electronic Supplementary Material (ESI) for RSC Advances.
This journal is © The Royal Society of Chemistry 2018

Accurate potential energy surfaces for the first two lowest electronic states of Li (2p) + H_2 reaction

Liwei Fu, Dequan Wang, Xuri Huang
Laboratory of Theoretical and Computational Chemistry,
Institute of Theoretical Chemistry,
Jilin University,
Changchun, People's Republic of China
E-mail: dequan_wang@jlu.edu.cn

Attachment files

Figure 1A: 1
Figure 2A:.................................... 2
Figure 3A: 3
Figure 4A: 4
Figure 5A: 5
Figure 6A: 6

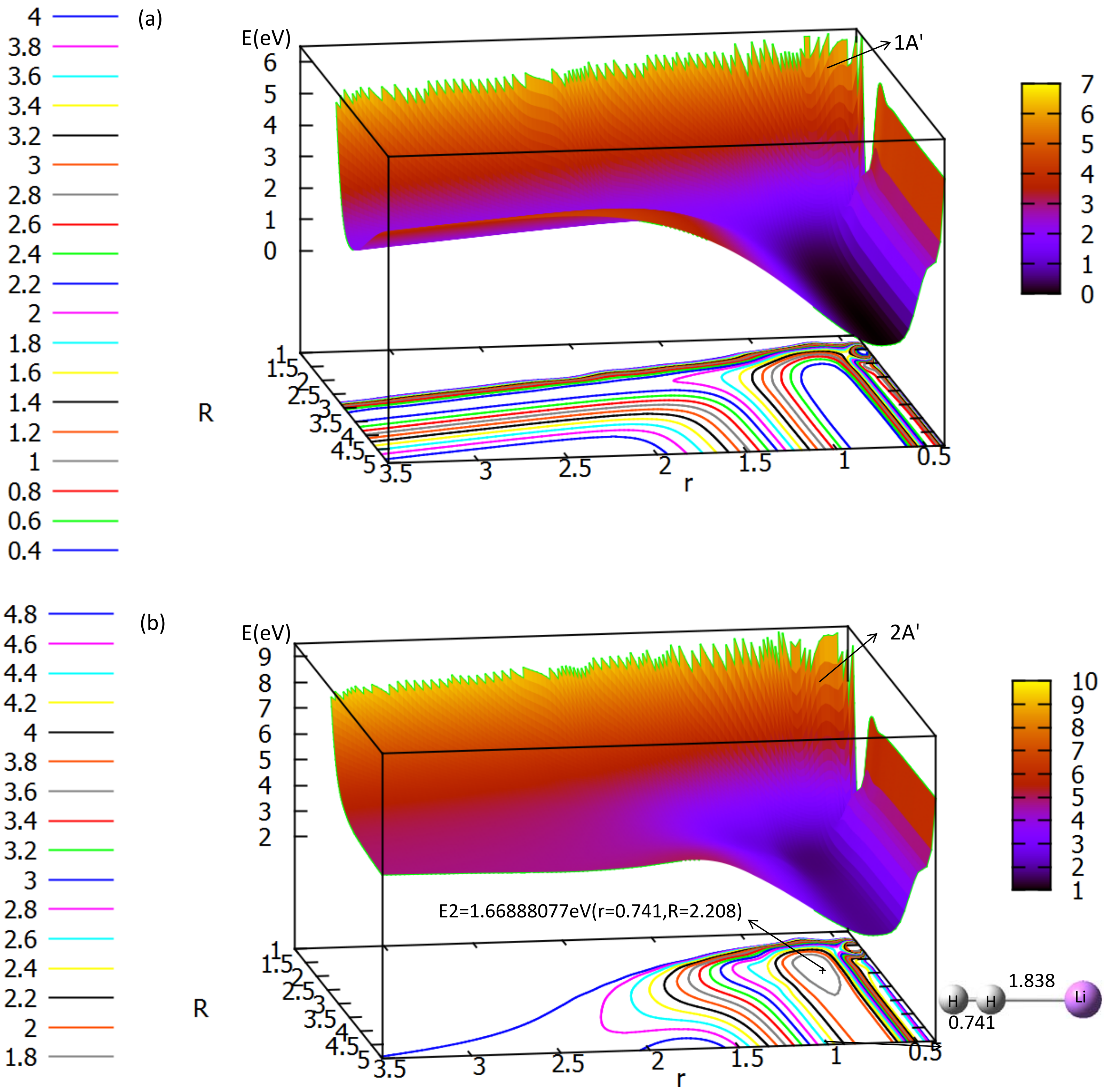

Figure 1A: Separated plotting the ground state (1A') and the first execited state (2A') PESs at θ=0° in Jacobi coordinate.

.

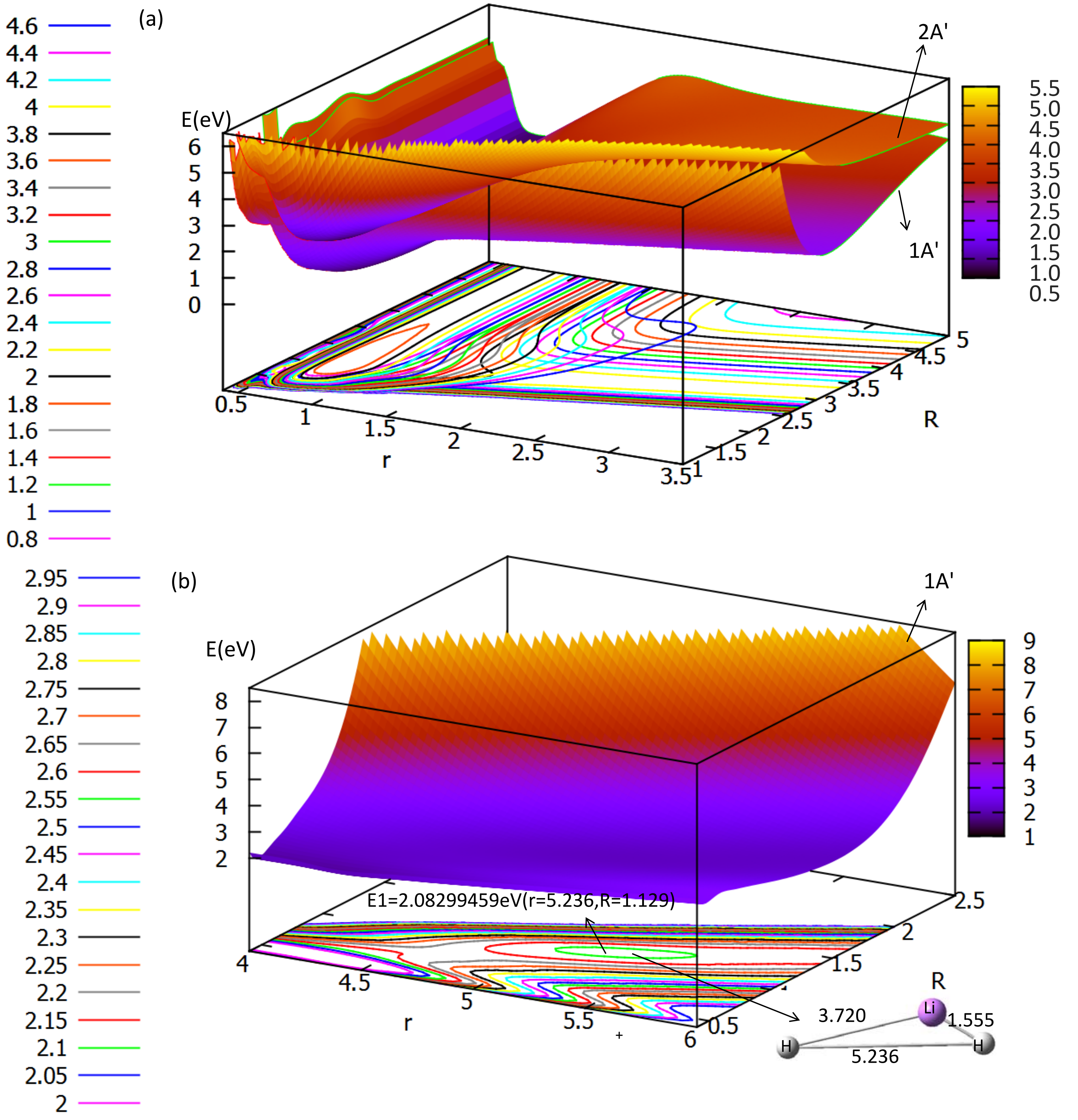

Figure 2A: Two potential energy surfaces (in eV) and contour plots of the potential energy surface as a function of distances r and R (in Ǻ) at θ=15°.

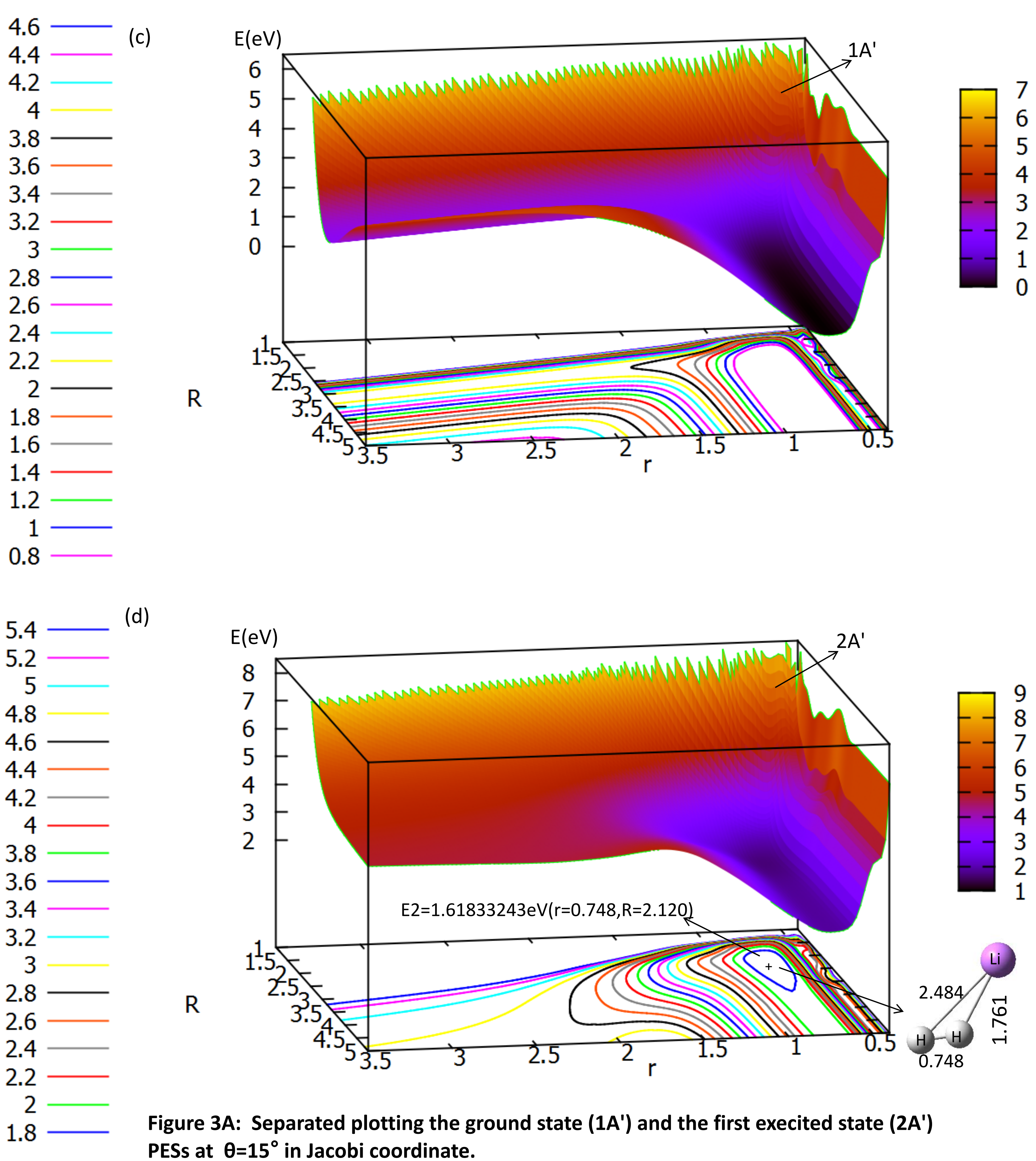

Figure 3A: Separated plotting the ground state (1A') and the first execited state (2A') PESs at θ=15° in Jacobi coordinate.

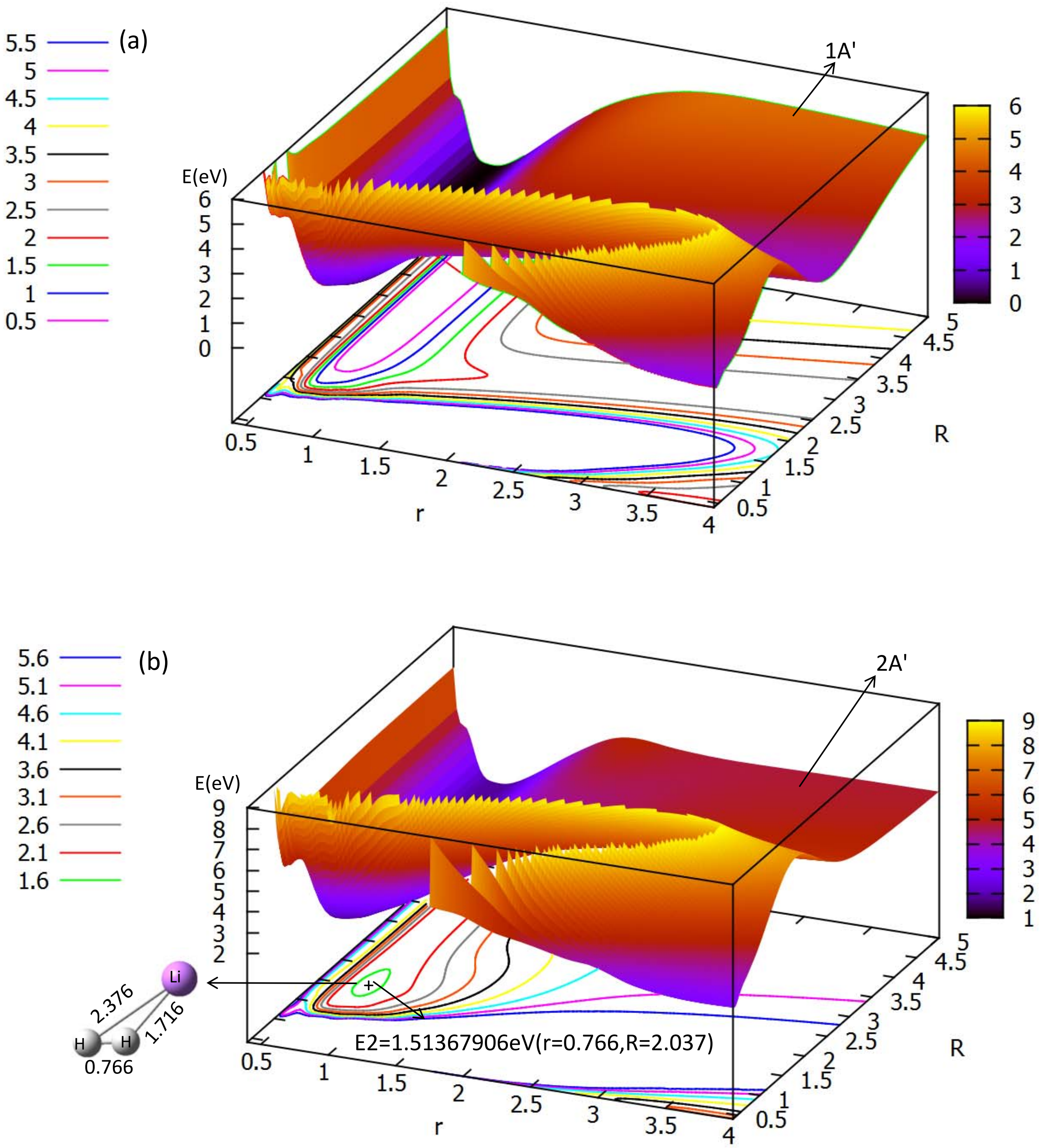

Figure 4A: Separated plotting the ground state (1A') and the first execited state (2A') PESs at θ=30° in Jacobi coordinate.

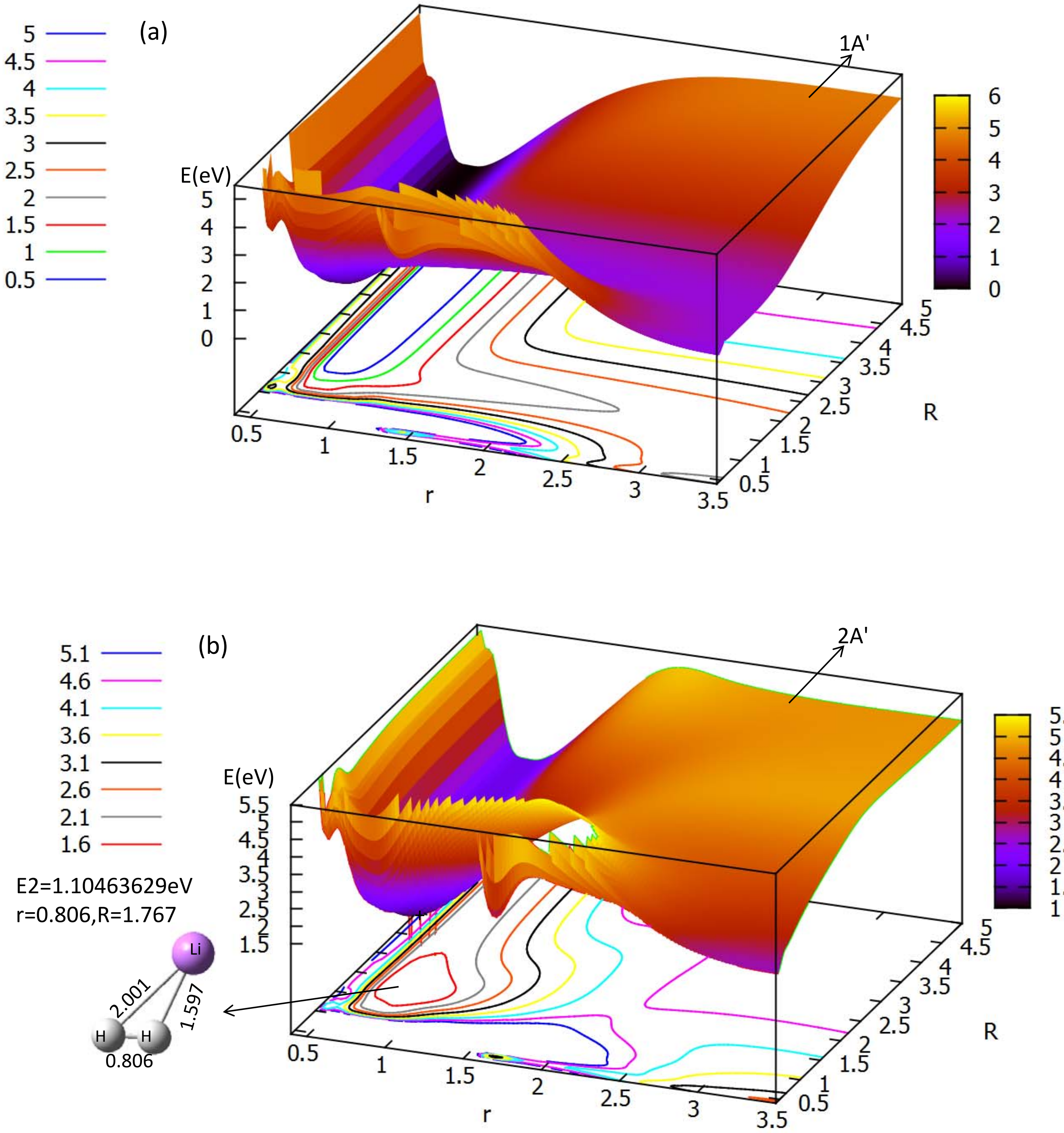

Figure 5A: Separated plotting the ground state (1A') and the first execited state (2A') PESs at θ=60° in Jacobi coordinate.

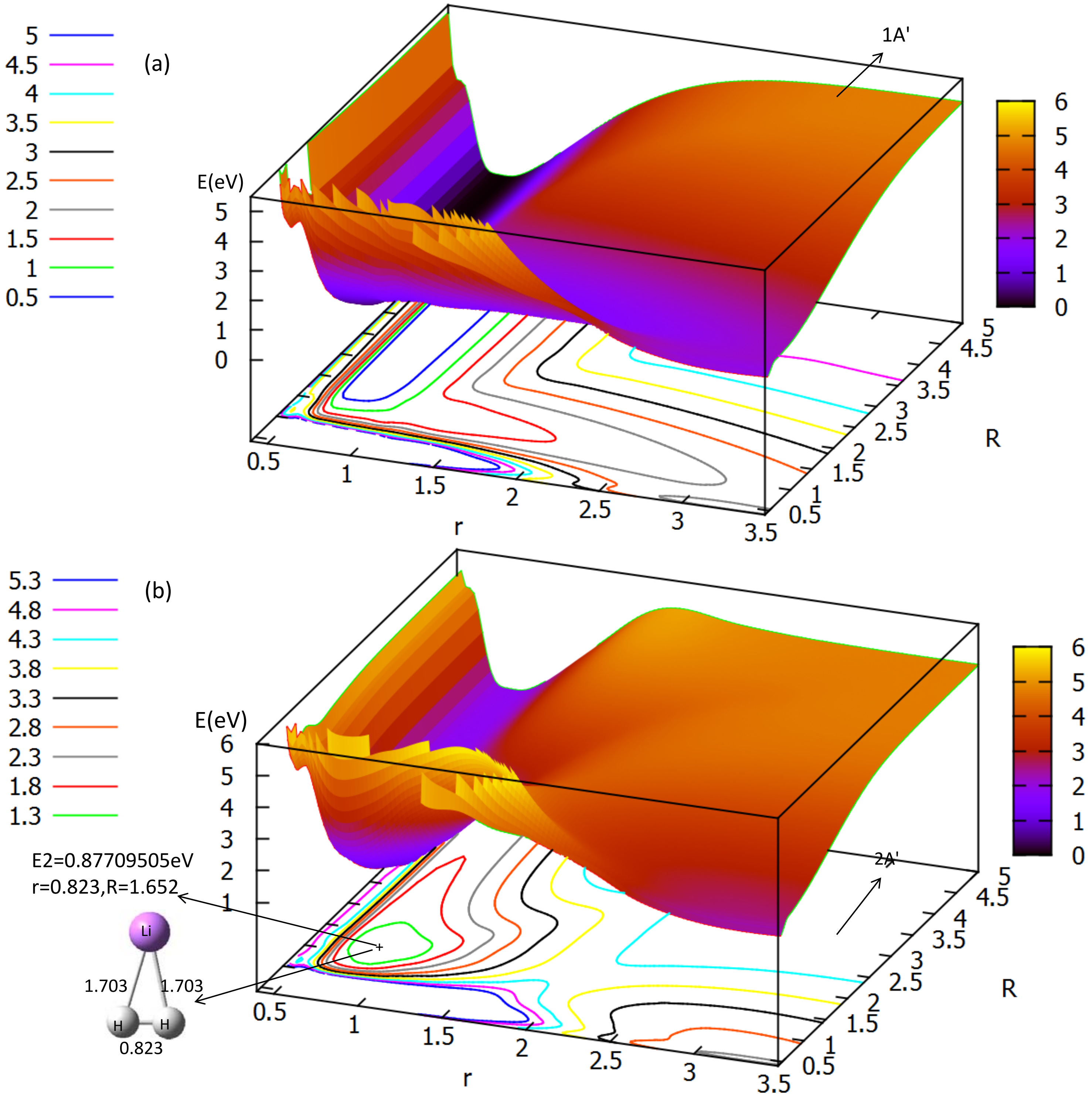

Figure 6A: Separated plotting the ground state (1A') and the first execited state (2A') PESs at θ=90° in Jacobi coordinate.
